# Canadian chronic kidney disease clinics: a national survey of structure, function and models of care

**DOI:** 10.1186/s40697-014-0029-2

**Published:** 2014-11-18

**Authors:** Adeera Levin, Soroka Steven, Allu Selina, Au Flora, Gil Sarah, Manns Braden

**Affiliations:** University of British Columbia, Vancouver, Canada; Dalhousie University, Halifax, Canada; Departments of Medicine and Community Health Sciences, University of Calgary, Calgary, Canada; CAnadian KidNey KNowledge TraNslation & GEneration NeTwork (CANN-NET), Calgary, Canada

## Abstract

**Background:**

The goals of care for patients with chronic kidney disease (CKD) are to delay progression to end stage renal disease, reduce complications, and to ensure timely transition to dialysis or transplantation, while optimizing independence. Recent guidelines recommend that multidisciplinary team based care should be available to patients with CKD. While most provinces fund CKD care, the specific models by which these outcomes are achieved are not known. Funding for clinics is hospital or program based.

**Objectives:**

To describe the structure and function of clinics in order to understand the current models of care, inform best practice and potentially standardize models of care.

**Design:**

Prospective cross sectional observational survey study.

**Setting, Patients/Participants:**

Canadian nephrology programs in all provinces.

**Methods and Measurements:**

Using an open-ended semi-structured questionnaire, we surveyed 71 of 84 multidisciplinary adult CKD clinics across Canada, by telephone and with written semi-structured questionnaires; (June 2012 to November 2013). Standardized introductory scripts were used, in both English and French.

**Results:**

CKD clinic structure and models of care vary significantly across Canada. Large variation exists in staffing ratios (Nephrologist, dieticians, pharmacists and nurses to patients), and in referral criteria. Dialysis initiation decisions were usually made by MDs. The majority of clinics (57%) had a consistent model of care (the same Nephrologist and nurse per patient), while others had patients seeing a different nephrologist and nurses at each clinic visit. Targets for various modality choices varied, as did access to those modalities. No patient or provider educational tools describing the optimal time to start dialysis exist in any of the clinics.

**Limitations:**

The surveys rely on self reporting without validation from independent sources, and there was limited involvement of Quebec clinics. These are relative limitations and do not affect the main results.

**Conclusions:**

The variability in clinic structure and function offers an opportunity to explore the relationship of these elements to patient outcomes, and to determine optimal models of care. This list of contacts generated through this study, serves as a basis for establishing a CKD clinic network. This network is anticipated to facilitate the conduct of clinical trials to test novel interventions or strategies within the context of well characterized models of care.

**Electronic supplementary material:**

The online version of this article (doi:10.1186/s40697-014-0029-2) contains supplementary material, which is available to authorized users.

## Abrégé structure

**Contexte:**

Les buts des soins aux patients atteints d’insuffisance rénale chronique (IRC) sont de retarder la progression de l’insuffisance rénale terminale, de restreindre les complications associées aux maladies concomitantes, de même que d’assurer une transition opportune vers la dialyse ou la transplantation, tout en optimisant l’indépendance. Les lignes directrices récentes recommandent des soins prodigués par une équipe multidisciplinaire aux patients atteints d’IRC. Bien que la plupart des provinces financent les soins aux patients atteints d’IRC, les modèles utilisés pour obtenir ces résultats ne sont pas connus. Le financement de ces cliniques provient généralement des hôpitaux ou de programmes.

**Objectifs:**

La description de la structure et de la fonction des cliniques, afin de comprendre les modèles de soins actuels, d’établir des pratiques exemplaires, ainsi qu’à la fois de comprendre et possiblement de normaliser des modèles de soins.

**Type d’étude:**

Étude d’observation transversale et prospective.

**Contexte:**

Les programmes de néphrologie dans toutes les provinces canadiennes.

**Méthodes:**

En nous basant sur l’infrastructure établie par le Canadian Kidney Knowledge Translation and Generation Network (CANN-NET), nous avons passé en revue 71 des 84 programmes, par l’entremise d’un questionnaire semi-structuré à réponses libres, soumis aux cliniques multidisciplinaires de soins aux patients atteints d’IRC de partout au Canada, par voie téléphonique et écrite, entre juin 2012 et novembre 2013. Un texte de présentation décrivait CANN-NET et exposait l’objet du sondage. Une infirmière francophone spécialisée dans les soins aux patients atteints d’IRC a traduit en français le texte de présentation, la lettre, de même que les questions, au bénéfice des sites francophones.

**Résultats:**

Il existe des variations importantes entre la structure et les modèles de soins des régions et des provinces canadiennes. On note une variation considérable entre les ratios de dotation en personnel, en ce qui concerne les néphrologues, les nutritionnistes, les pharmaciens et les infirmières, de même qu’entre les critères d’orientation. Les décideurs en matière d’amorce de la dialyse étaient généralement des MD, bien que dans quelques cas, une approche basée sur la concertation était privilégiée. La majorité des cliniques (57 %) suivaient un modèle cohérent de soins par un même néphrologue et une même infirmière pour tous les patients, alors que d’autres décrivaient des variations dans la continuité des soins, parmi lesquelles des patients qui étaient suivis par différents néphrologues et différentes infirmières d’une visite à l’autre. Les cibles concernant diverses modalités variaient beaucoup, de même que l’accès à ces modalités. Aucune des cliniques de soins aux patients atteints d’IRC n’était dotée de ressources ou d’outils informant les patients et les prestataires de soins sur le moment optimal pour amorcer une dialyse.

**Conclusion:**

La viabilité de la structure et de la fonction de la clinique offre l’occasion d’explorer la relation entre ces éléments et les résultats pour le patient, de même que de déterminer les modèles optimaux de soins aux patients. La présente étude, ainsi que la liste des ressources qui y sont énumérées, servent de base pour l’établissement d’un réseau de cliniques de soins aux patients atteints d’IRC, afin d’étudier de près ces questions et de procéder à de futurs essais cliniques. Les résultats du sondage peuvent servir à établir les priorités parmi les activités de transfert des connaissances, particulièrement celles qui entourent le moment d’amorcer la dialyse et les choix qui concernent les modalités de la dialyse. On prévoit que ce réseau facilitera la réalisation d’essais cliniques visant à tester de nouvelles interventions ou de nouvelles stratégies dans le contexte de modèles de soins bien caractérisés.

**Limites de l’étude:**

Il nous a été impossible de vérifier les renseignements qui concernent ceux qui n’ont pas répondu. Il y a eu des différences entre les réponses parmi les provinces, particulièrement au Québec, malgré les versions traduites en français des questionnaires. Le contexte dans lequel ces cliniques évoluent n’a pas été documenté : la proximité avec des cliniques d’évaluation des transplantations, les éducateurs en dialyse péritonéale, de même que les installations d’insertion de cathéter de dialyse péritonéale au chevet du patient peuvent s’avérer des facteurs déterminants dans les résultats pour les patients. La structure de financement des cliniques n’a pas non plus été déterminée, de même que leur emplacement dans les hôpitaux, tant dans les établissements pour les patients externes qu’en dehors du contexte hospitalier.

## What was known before

There is little in the literature about the specific structures of the multidisciplinary care clinics for patients with chronic kidney disease. There is a large literature describing general chronic disease management, and the need for multidisciplinary care in CKD. No information about the organization of CKD care before dialysis in Canada exists.

## What this adds

This paper describes the variability in various components of CKD clinic structures across Canada. The staffing models of health professionals to patients vary, as does the model of care. This descriptive study is a first essential step in improving understanding of the relationship of CKD models of care to patient outcomes.

## Background

Patients with chronic kidney disease (CKD) do not always receive care consistent with guidelines, in part due to complexities in CKD management, lack of randomized trial data to inform care, and a failure to disseminate best practice. Published guidelines from national and international groups describe the importance of access to multidisciplinary care teams for patients with severe CKD [1–4] _ENREF_1, based in part on successful models in other complex diseases [5–8], and in part based on data suggesting that such multidisciplinary care slows progression to kidney failure and is associated with optimal preparation for dialysis [9–12].

The goals of CKD care are to improve patient outcomes, delay CKD progression towards end-stage renal disease, reduce the risk of cardiovascular disease and other complications associated with CKD, and prepare individuals and their families for transition to dialysis, transplantation or conservative care, as appropriate [13–15]. Increasing emphasis has been placed on the importance of education and preparation of patients so as to optimize the uptake of home-based or independent therapies, as these have been associated with improved outcomes and lower costs [16,17]. Despite these initiatives, and the existence and funding of CKD clinics in nearly all Canadian jurisdictions, there have been no standardized definitions or structure of CKD clinics, nor any metrics by which to evaluate these clinics within the nephrology communities.

In Canada, CKD clinics are usually funded from provincial or regional health care dollars, though in some areas of Canada, funding still flows through hospital budgets. In the era of increasing accountability, evidence-informed care, and the potential relationship of different CKD clinic models of care to patient outcomes and costs, it is critical that we understand current CKD care models in place across Canada. This information will be used to inform best practices across the country, with the potential for standardization.

CANN-NET (CAnadian KidNey KNowledge TraNslation & GEneration NeTwork) is a multi-disciplinary network of clinicians, researchers and decision makers in Nephrology that tackles priorities that were identified based on an assessment of gaps in the care of people with kidney disease, supplemented with a survey of heads of Canadian renal programs. These include priorities for new clinical practice guidelines and knowledge translation activities [18], as well as priority areas for new research. With funding from the Canadian Institutes of Health and the Kidney Foundation of Canada, CANN-NET was developed in partnership with the Canadian Society of Nephrology to improve the care and outcomes of patients with CKD. A selection of the top priorities were finalized at a face-to-face meeting of knowledge users in March 2011, and included timing of dialysis initiation and increasing appropriate use of home dialysis. Given that these key priorities are two activities that are closely linked to the CKD care that is provided in multidisciplinary CKD clinics, an improved understanding of CKD clinic function and structure was noted to be important. Since the long-term goal of CANN-NET is to improve CKD care and outcomes across its continuum, the development of a network of key contacts at multidisciplinary CKD clinics across Canada was also noted to be important. As a preliminary step towards this, we surveyed CKD clinics across Canada to understand more about current practice and resources available within these clinics.

## Methods

An open-ended semi-structured questionnaire, developed by the CANN-NET Knowledge Translation (KT) Committee, was administered to multidisciplinary adult CKD clinics across Canada between June 2012 and November 2013 (see Additional file 1). In addition to the questionnaire, an introductory script was used to introduce CANN-NET and highlight the purpose of the survey. A French speaking CKD nurse translated the introductory script, the letter and survey questions into French for use in French-speaking sites.

The process of contacting and conducting questionnaires was as follows. Members of the nephrology community that were part of CANN-NET, or who had worked with members of CANN-NET, were contacted to determine names of the multidisciplinary adult CKD clinics in their areas, regions, or provinces, as well as the name and contact numbers of the medical or nursing director of the CKD clinic. Surveys were then conducted by phone by CANN-NET staff after verifying the correct person at each site. To maximize the number of clinics contacted, leaders of the nephrology community across the country were engaged to assist the research team by also contacting potential survey participants.

The survey included 20 questions and took between 20–40 minutes to complete when conducted by telephone. Responses were noted by the CANN-NET staff and transcribed into an Excel spreadsheet. For information not immediately available, additional information was provided by email. In a minority of cases, CKD clinic leads provided information on each question within a word document, rather than by telephone. In these cases, any information that was unclear was confirmed by email, with confirmation of any responses that were unclear. Surveys could be conducted or completed in both English and French. Ethics approval was obtained by the Conjoint Health Research Ethics Board at the University of Calgary.

## Results

**Figure 1:**
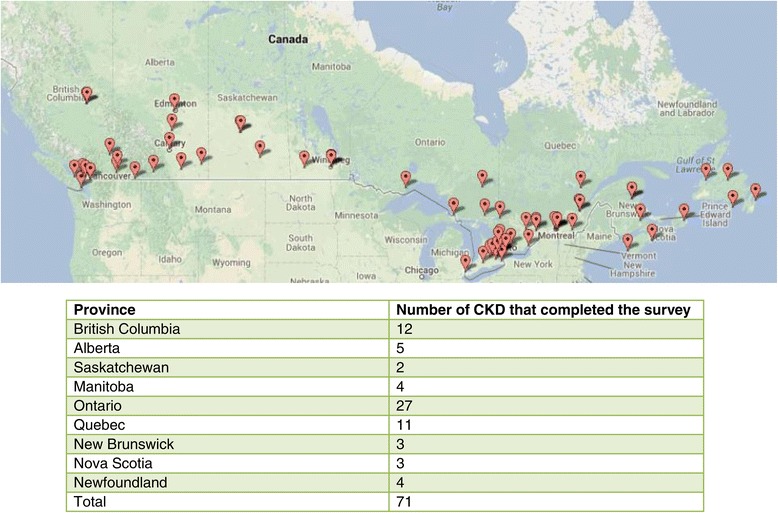
**71 Completed multidisciplinary CKD Clinics surveys by province.** Describes the location of clinics surveyed throughout Canada by province: note that all provinces were included, and multiple clinics in each province participated both from academic and non academic centres.

Between June 2012 and November 2013, 84 multidisciplinary adult CKD clinics across Canada were contacted and 71 completed the survey, 48 surveys were conducted via telephone. Of the 71 completed surveys, 23 were in Western Canada, 27 in Ontario, 11 in Quebec, and 10 in the Atlantic Provinces (Figure 1). Of the 71 surveys, one third were conducted with medical leads or physicians and the remainder were completed by nursing or administrative CKD clinic managers.

### Variation in size and staffing ratios of CKD clinics

**Figure 2:**
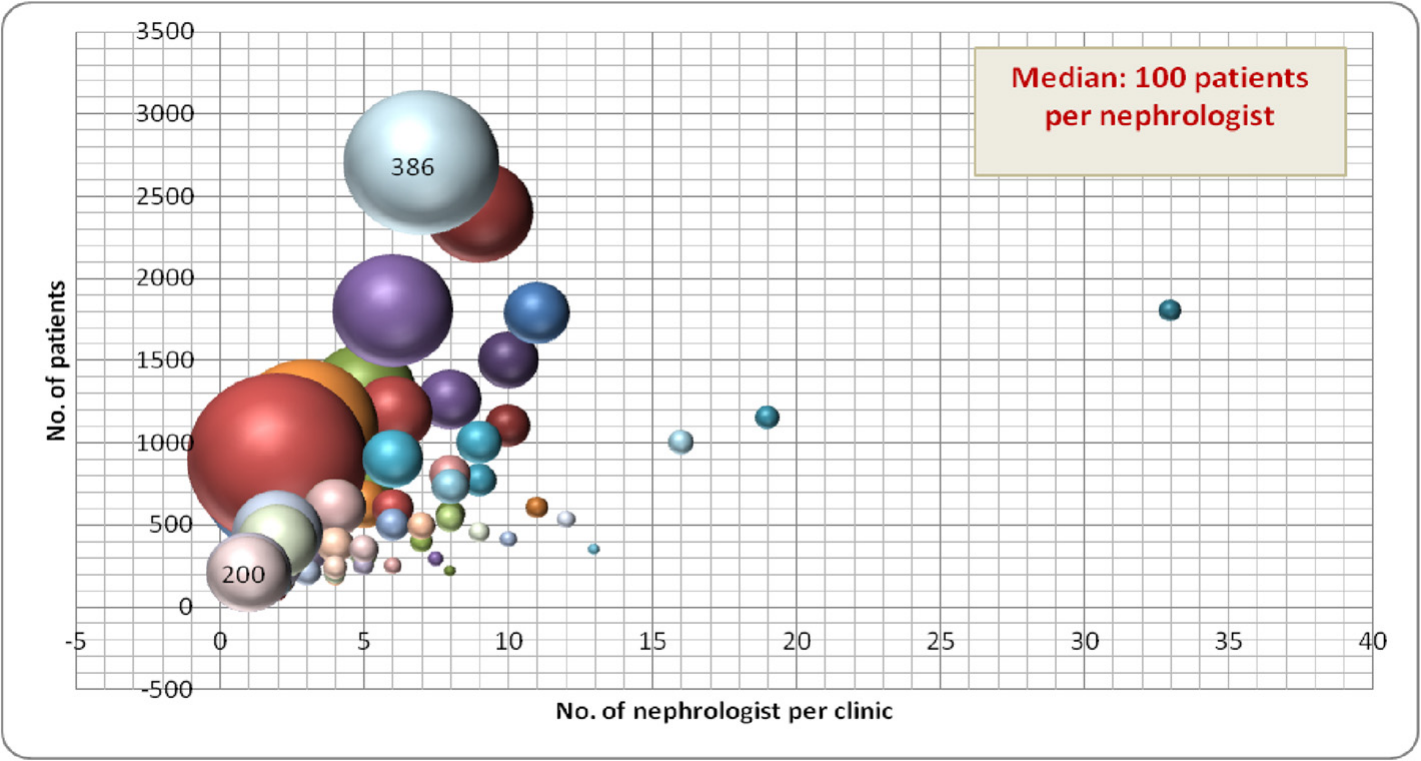
The number of patients and nephrologists in each multidisciplinary CKD clinic. *Each bubble represents the ratio of the number of patients/number of nephrologists (larger bubbles represent a larger ratio). Note the variability in the ratios. See text for possible explanations.

**Figure 3:**
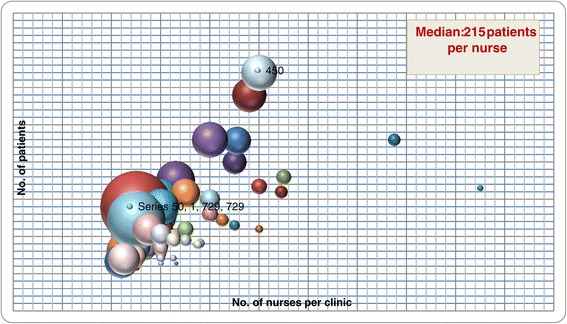
The number of patients and nurses in each multidisciplinary CKD clinic. *Each bubble represents the ratio of the number of patients/number of nurses (larger bubbles represent a larger ratio).

CKD clinics cared for between 26 and 2700 patients, and were staffed with between 1 and 33 nephrologists. The staffing ratio of patients to nephrologists ranged from 26 to 450 across the clinics (Figure 2). The staffing ratio of patients to CKD clinic nurses also varied from 9 to 900 (Figure 3). Almost all clinics employed dietitians 67/71, or 94%), pharmacists (50/71, or 70%) and social workers (65/71, or 91%).

### Referral criteria

Of the clinics that reported having eGFR referral criteria (35/71, or 48%), the majority (25/35, or 71%) indicated that referral was based on eGFR <30 mL/min/1.73 m2. The remaining indicated that the eGFR referral range was between 30 and 60 mL/min/1.73 m2 (14%).

### Dialysis modalities provided and modality education in the clinics

**Figure 4:**
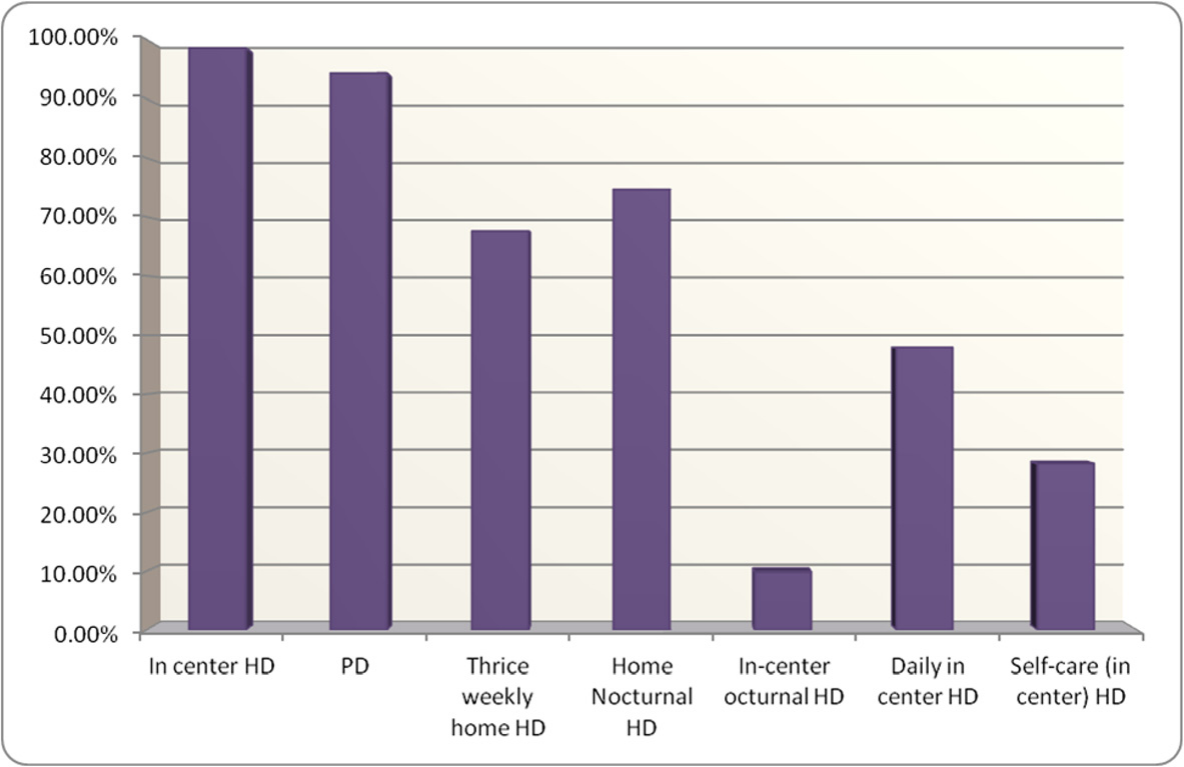
**The percentage of clinics that work within renal programs which offer specific different types of dialysis.** Not all CKD clinics are co-located within renal programs that offer all of the different modalities.

All clinics operated within the context of a renal program that offered in-center hemodialysis (99%), and nearly all programs offered peritoneal dialysis (94%), home thrice weekly hemodialysis (66%), and home nocturnal hemodialysis (73%) (Figure 4). Daily in-center hemodialysis, self-care in center hemodialysis, and in-center nocturnal hemodialysis were offered less frequently (Figure 4). 73% of programs (52 CKD clinics) had a policy in place whereby all patients were assessed for home dialysis. Of the remaining 19 CKD clinics, only one program did not offer home dialysis at all. The rest of the CKD clinics indicated that patients were assessed for home dialysis even though no policy was in place per se. While all programs noted that they offer dialysis modality education, 41% offered this during regular clinic visits or through distribution of educational materials alone, while 59% offered group dialysis modality education. Only 32% of programs employed a dedicated dialysis modality coordinator, though programs noted that individual CKD clinic nurses often filled this role (Table 1).

**Table 1:** **Key characteristics of the multidisciplinary clinics**

**Referral criteria to CKD clinic**		
**Referral criteria?**	57/71	**80.28%**
**eGFR cut off as part of the criteria?**	35/57	**61.40%**
**Management of patients nearing dialysis initiation**		
**Policy whereby all patients assessed for home dialysis**	52/71	**73.24%**
**All patients offered dialysis modality education?**	71/71	**100.00%**
**Dialysis modality education**		
**During clinics visits or through educational materials**	29/71	**40.85%**
**Group dialysis modality education (or combination)**	42/71	**59.15%**
**Decision to initiate dialysis made in conjunction with regular multidisciplinary team meetings**	27/71	**38.03%**
Dedicated dialysis modality coordinator?*	23/71	**32.39%**
**Do they see all patients who are approaching the need for dialysis?**	18/23	**78.26%**

Note that there is variability in referral criteria and in dialysis initiation criteria.

*Though individual nurses may fulfill this role in some centers.

### Modality targets, timing of dialysis initiation and role of nephrologist in decision making

#### Modality targets

With respect to home dialysis targets, 73% of programs noted that their renal program operated within an environment where they were aware of a target for home dialysis use. For 42% of these programs, the target is based on the proportion of prevalent home dialysis patients. In 48% of programs, the target was based on both incident and prevalent home dialysis use, and for 7% of clinics, the target was based on incident home dialysis use only (Table 2).

**Table 2:** **Multidisciplinary CKD clinic staff opinions for program improvement, generated from open ended questions**

**Centers are aware of home dialysis target 52/71 73.23%**		
**Target is based on prevalent patients**	22/52	42.31%
**Target is based on incident patients**	4/52	7.69%
**Target is based on both incident and prevalent patients**	25/52	48.08%

Note that this is a list compiled from individual comments, and grouped according to themes.

#### Timing of dialysis initiation

25 clinics (35%) stated that eGFR levels alone generally led to dialysis initiation in out-patients with progressive CKD, but clinics often noted that other factors, including symptoms, were important in the decision to initiate dialysis. Of the 25 clinics noting that an eGFR level was important on its own in leading to dialysis initiation, 40% (10) stated a threshold level of <10 mL/min/1.73 m2, while 20% (5) stated a threshold level of 10–15 mL/min/1.73 m2 An additional 8 clinics indicated dialysis was sometimes initiated above 10 mL/min/1.73 m2. Two clinics did not answer the question.

#### Decision making

**Figure 5:**
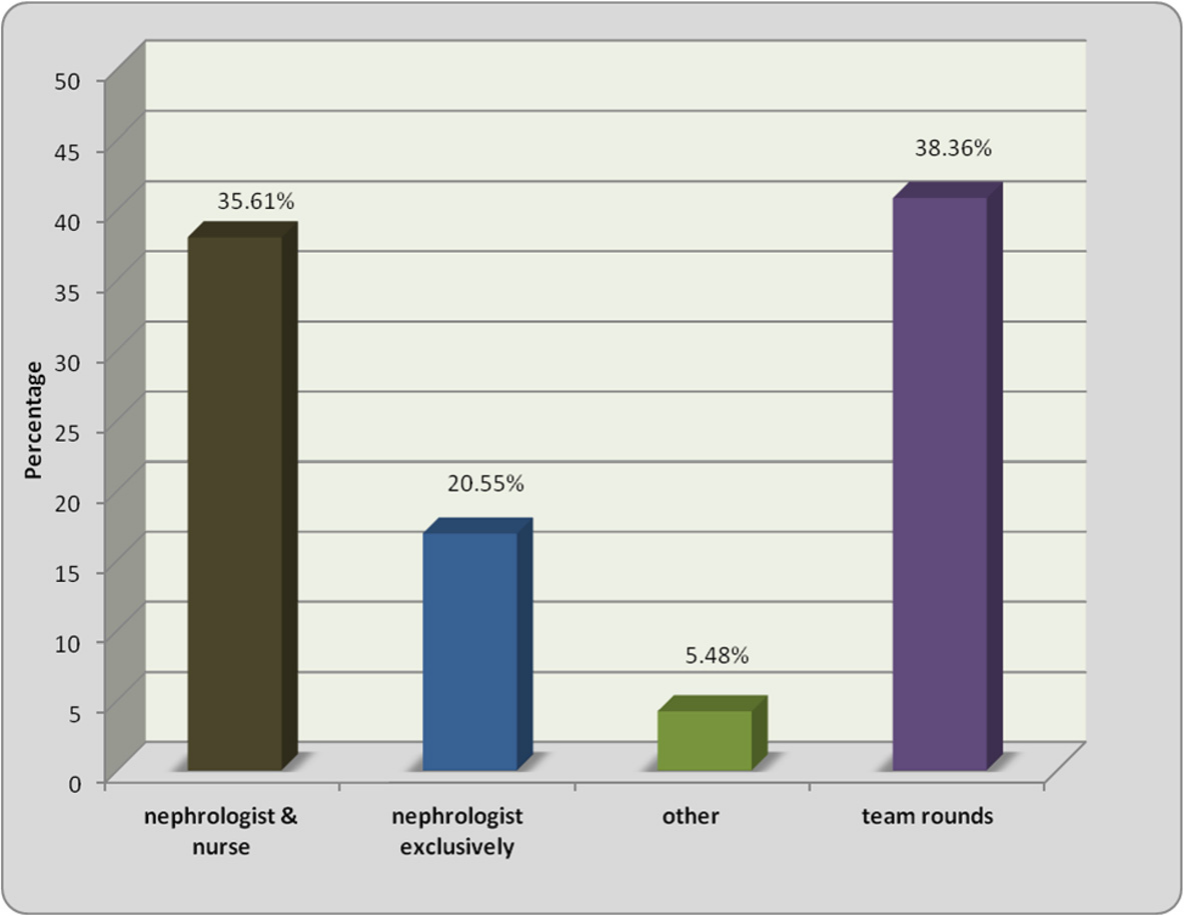
**Decision to start on patient on dialysis - reviewed/decision made by whom?** This figure describes the variation in decision makers in CKD clinics, with equal proportions of either nephrologists alone, nurses alone or multidisciplinary teams being responsible for dialysis initiation decisions.

Nephrologists were felt to have the main role in the decision-making process of when to initiate dialysis. Only 38% of CKD clinics currently have a multidisciplinary team meeting where the timing of dialysis initiation is discussed (Figure 5).

### Model of patient care by nephrologists and nurses within the CKD clinic

**Figure 6:**
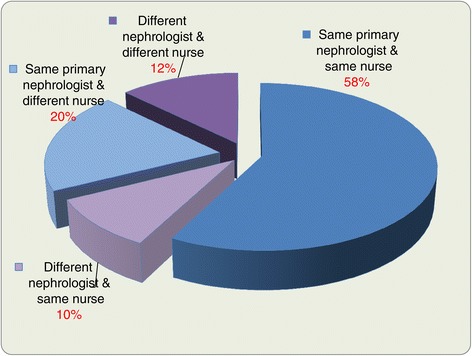
**Model of Nephrologists and nurse care within the CKD clinic.**

The majority of clinics (57%) cared for patients using the same nephrologist and nurse. In a small percentage of clinics (12%), patients would see a different nephrologist and a different nurse at each visit (Figure 6).

### Challenges, suggestions for improvements and strengths of existing programs for CKD care as noted by respondents

Respondents provided their opinions on where improvements could be made to improve patient outcomes. The most common issues that were raised included that more staff, space and improved clinic processes were required (Table 3). Having resources to help emphasize home-based therapies more consistently was also cited as a problem.

**Table 3:** **Multidisciplinary CKD Clinic staff opinions about things that could be improved in their renal programs**

**Suggestions for improvement (overall themes)**	
**1**	Request for more staff (or more time from existing staff) (nurses, nephrologists, experts, clerical staff, data leads, etc.)
**2**	Need for more space
**3**	Need for more/improved/standardized teaching or educational aids/tools
**4**	Need for better CKD clinic processes (better flow of patient referral, standardized guidelines, decrease wait times, offer telehealth, offer clinical pathway, timely insertion of catheters, better communication, team model, etc.)
**5**	Encourage home therapies, more dialysis options, support for dialysis for patients in rural communities, early identification of patients for home therapies, patient council, etc.
**6**	Early outreach/referral of patients, preventative programs

Note that this is a list compiled from individual comments, and grouped according to themes.

Respondents also noted that several things were working well within their clinics (Table 4), mostly centering around activities focused on improving outcomes, patient experience and team functioning to achieve goals of care. They included initiatives around conservative care, development of in-house teaching and educational tools specific to the population/clinic, increased outreach for early CKD detection, the implementation of “buddy system” (dedicated nurse assigned to patients working with the nephrologists); nurse-led tele-health clinic in a remote areas, patient support groups, and the inclusion of patients in continuous quality improvement initiatives.

**Table 4:** **Multidisciplinary CKD clinic staff opinions about things that worked well in their renal programs: what worked well in the care of patients with kidney disease**

**What worked well in the care of patients with kidney disease (overall themes)**	
1.	Starting new initiatives around conservative care as more people are choosing this option.
2.	Developing in-house teaching and educational tools that are specific to their population/clinic
3.	Traveling band of health care providers who provide outreach in the community for early CKD detection
4.	The “buddy system” (dedicated nurse assigned to a patient who worked with the nephrologists) allows for greater interaction with the patient and builds trust.
5.	A nurse-led telehealth clinic in a remote area allows CKD patients to stay in their communities while being followed by the multidisciplinary team in a large center.
6.	Patient support groups (where patients support new patients)
7.	Having a strong/well-resourced team of unit clerks and administrative staff at the CKD clinics play a large and important role in clinic activities.
8.	Inclusion of CKD patients in QI project teams
9.	Having a strong multidisciplinary team, dedicated and supportive staff, and good teamwork despite limited resources
10.	Timely and appropriate vascular access

Note that this is a list compiled from individual comments, and grouped according to themes.

## Discussion and conclusions

This study represents the first attempt at describing CKD clinic structures across Canada, using a standard method of data collection. This extensive and comprehensive survey highlights significant variation in CKD clinic structure and models of care across regions and provinces in Canada. There are differences with respect to staffing ratios, eligibility criteria for being assessed for home dialysis, access to dedicated educational resources, and indications for dialysis initiation. Despite access to a variety of educational materials, none of the CKD clinics had patient or provider educational resources/tools educating patients and providers on the optimal time to start dialysis.

The variation in size and staffing ratios may be related to ‘entry criteria’ and purpose of the CKD clinics (general nephrology combined with dialysis preparation clinics versus dedicated dialysis preparation clinics), or simply to funding models and patient selection. While some had referral criteria, not all did, and there were no consistent criteria across the country. Despite increasing emphasis on home-based therapies over the last five years in particular, not all programs offer all home based options, and not all clinics are operating within full service programs.

As in all studies, there are strengths and limitations to this survey. While comprehensive information was collected from a large number of diverse multidisciplinary CKD clinics across Canada, we were not able to ascertain information from those who did not respond: thus even the characteristics of the non- responders are not possible. Despite significant attempts to contact all clinics in all provinces, there was differential response by province, particularly in Quebec, despite translation of questionnaires into French. This survey relied upon respondents’ perception of clinic structure and function, and may not reflect actual CKD clinic function and structure, though the respondents were generally all medical or nursing clinic leads. Despite knowing the numbers of health care professionals ‘available’ in the CKD clinics, we do not know the frequency or nature of interactions between them and the patients. We do not have information that links CKD clinic structure and function to patient outcomes, such as uptake of home based therapies, planned starts on dialysis, pre-emptive transplantation, and selection of conservative supportive care instead of renal replacement options. Furthermore, we do not have any information on the patient experience in these different CKD clinic structures. Nonetheless, none of these limitations impact the value of the data collected, nor diminish the utility of the survey results in establishing a baseline fundamental understanding of how multidisciplinary CKD clinics currently function.

The context in which the specific clinics operate was not captured: whether there are adjacencies to transplant assessment clinics, PD educators, bedside PD catheter insertion facilities may all be important determinants of patient outcomes. The funding structure of the clinics was also not determined, nor their location within hospitals, in outpatient facilities or outside of hospital structures.

Further research is needed to understand how CKD clinic structure/model of care influences patient care and outcomes. Understanding this would have important policy implications. For example, is there one (or more) model of care that results in optimal outcomes for patients and what is the impact on costs? What factors or elements make one clinic structure more efficient when compared to a similar sized clinic? What is the impact of provincial health care structures on clinic functioning and patient outcomes? With this information, we may be better informed to advise administrators and clinicians as to best practices and recommend models of care. Furthermore, we may be able to gain valuable insights by comparing these results to those of other countries with similar health care systems (UK and Australia). To our knowledge, there have not been any formal assessments, using a uniform taxonomy to describe clinic structure and function, as part of CKD disease management, in any other jurisdictions. Lastly, how and if changes in models of care impact outcomes is not known, and would also be an important area of future research.

This survey, and the list of contacts generated herein will facilitate the development of a national network of CKD clinics. Including patients and their families in this next phase of work will be imperative to ensuring that we ask and answer the right questions. Such a network could use the results from this survey, as well as information on local practice to prioritize knowledge translation activities that the CANN-NET Knowledge User and Knowledge Translation Committees can pursue, particularly around the timing of dialysis initiation and dialysis modality choices. The current findings also encourage us to address additional questions based on structure and function, strengthening the network. This network is anticipated to facilitate the conduct of clinical trials to test novel interventions or strategies within the context of well characterized models of care.

## Additional file

Additional file 1:
**Survey.**

## References

[1] Kidney Disease: Improving Global Outcomes (KDIGO) CKD Work Group (2013). KDIGO 2012 clinical practice guideline for the evaluation and management of chronic kidney disease. Kidney Int Suppl.

[2] Levin A, Hemmelgarn B, Culleton B, Tobe S, McFarlane P, Ruzicka M, Burns K, Manns B, White C, Madore F, Moist L, Klarenbach S, Barrett B, Foley R, Jindal K, Senior P, Pannu N, Shurraw S, Akbari A, Cohn A, Reslerova M, Deved V, Mendelssohn D, Nesrallah G, Kappel J, Tonelli M (2008). Guidelines for the management of chronic kidney disease. CMAJ.

[3] Burden R, Tomson C (2005). Guideline development committee, joint specialty committee on renal disease of the Royal College of Physicians of London the Renal association. Identification, management and referral of adults with chronic kidney disease: concise guidelines. Clin Med.

[4] ᅟ (2014). KHA - CARI Guidelines.

[5] Senior PA, MacNair L, Jindal K (2008). Delivery of multifactorial interventions by nurse and dietitian teams in a community setting to prevent diabetic complications: a quality- improvement report. Am J Kidney Dis.

[6] Adams SG, Smith PK, Allan PF, Anzueto A, Pugh JA, Cornell JE (2007). Systematic review of the chronic care model in chronic obstructive pulmonary disease prevention and management. Arch Intern Med.

[7] Sunaert P, Bastiaens H, Feyen L, Snauwaert B, Nobels F, Wens J, Vermeire E, Van Royen P, De Maeseneer J, De Sutter A, Willems S (2009). Implementation of a program for type 2 diabetes based on the chronic care model in a hospital-centered health care system: “the Belgian experience”. BMC Health Serv Res.

[8] To T, Cicutto L, Wajja A, McLimont S, Shahsavar A, Ghulmiyyah M (2006). Evaluation of a community-based primary care asthma pilot project. Am J Respir Crit Care Med.

[9] Johnson DW (2004). Evidence-based guide to slowing the progression of early renal insufficiency. Intern Med J.

[10] Nunes JAW, Wallston KA, Eden SK, Shintani AK, Ikizler TA, Cavanaugh KL (2011). Associations among perceived and objective disease knowledge and satisfaction with physician communication in patients with chronic kidney disease. Kidney Int.

[11] Mason J, Khunti K, Stone M, Farooqi A, Carr S (2008). Educational interventions in kidney disease care: a systematic review of randomized trials. Am J Kidney Dis.

[12] Devins GM, Mendelssohn DC, Barre PE, Taub K, Binik YM (2005). Predialysis psychoeducational intervention extends survival in CKD: a 20-year follow-up. Am J Kidney Dis.

[13] Wei SY, Chang YY, Mau LW, Lin MY, Chiu HC, Tsai JC, Huang CJ, Chen HC, Hwang SJ (2010). Chronic kidney disease care program improves quality of pre-end-stage renal disease care and reduces medical costs. Nephrology.

[14] Klang B, Bjorvell H, Clyne N (1999). Predialysis education helps patients choose dialysis modality and increases disease-specific knowledge. J Adv Nurs.

[15] Klang B, Bjorvell H, Berglund J, Sundstedt C, Clyne N (1998). Pre-dialysis patient education: effects on functioning and well-being in uraemic patients. J Adv Nurs.

[16] Klarenbach S, Manns B (2009). Economic evaluation of dialysis therapies. Semin Nephrol.

[17] Howard K, Salkeld G, White S, Mcdonald S, Chadban S, Craig JC, Cass A (2009). The cost- effectiveness of increasing kidney transplantation and home-based dialysis. Nephrology.

[18] Manns B, Barrett B, Evan M, Garg A, Hemmelgarn B, Kappel J, Klarenbach S, Madore F, Parfrey P, Samuel S, Soroka SD, Suri R, Tonelli M, Wald R, Walsh M, Zappitelli M, NeTwork FtCKKTaG: **Establishing a national knowledge translation and generation network in kidney disease: the CAnadian KidNey KNowledge TraNslation and GEneration NeTwork.** *CJKHD* 2014, **1**(2). doi:10.1186/2054-3581-1181-1182. 10.1186/2054-3581-1-2 PMC4346238 25780597

