# Supplementary material for: Canadian chronic kidney disease clinics: a national survey of structure, function and models of care
Source: Can J Kidney Health Dis. 2014 Nov 18;1:29. doi: 10.1186/s40697-014-0029-2 (PMC4349614; doi:10.1186/s40697-014-0029-2)
Supplement: Additional file 1: — Survey. [file 40697_2014_29_MOESM1_ESM.pdf]

Survey:

Date of telephone survey:
Time:
Name of the person being interviewed:
Details of CKD Clinic:
Person Conducting the interview:

Understanding the structure of CKD clinics in Canada

1. Do you have a multidisciplinary CKD or kidney care clinic? **Yes/No (collect name)**
2. How many multidisciplinary CKD clinics there are in your region?
3. a. How many Adult nephrologists practice in your multidisciplinary clinic?
   b. How many ADULT nephrologists practice in your local area (hospital, region, etc)?
4. Do all nephrologists refer patients with severe kidney disease to the multidisciplinary CKD clinics? **Yes/No**
5. Of the nephrologists in your region/hospital, do they use the resources at the multidisciplinary CKD clinic - **All of the resources for appropriate patients / Some of the resources for appropriate patients / None of the resources**
6. A. Do you have a co-leadership model for your CKD clinic? **How is the clinic directed? YES/NO (other)**
   B. Who is the medical, administrative, and nursing lead for your (admin and nursing lead may be the same person):

**OVERALL Renal Program**		
**Medical lead/Director**	**Administrative lead**	**Nursing lead**

**YOUR CKD Clinic**		
**Medical Lead/Director**	**Administrative lead**	**Nursing lead**

7. What type of dialysis does your local renal program offer? ***Tick where applicable…***

In Centre HD	
PD	
Home HD x 3/week	
Home Nocturnal HD	
In-center nocturnal HD	
Daily in center HD	
Self-Care (in centre)	

8. A. Does your lab report eGFR or just serum creatinine? **eGFR and creatinine / creatinine only**

B. Are their specific referral criteria for your clinic? **Yes/No (specify)**

C. Is there an eGFR cut off for referral to your CKD clinic? **Yes/No**

D. Are there other factors that influence referral to your CKD clinic? **Yes/No (specify)**

9. How large is your multidisciplinary CKD clinic?
   A. How many **patients (total)**
   B. How many **nurses** (FTE?)
   C. How many **dieticians** (FTE?)
   D. How many **pharmacists** (FTE)
   E. How many **social workers** work there?
   F. **Other** professionals (kinesiologist)?

10. **With respect to clinic visits, and how patients are cared for between clinic visits, what is the model of care with respect to the Nephrologist and the nurse? (see table):**

**Same primary nephrologist, same nurse**	**Same primary nephrologist, different nurses** (whoever is on clinics that day)	**Different nephrologist** (whoever is on clinics that day), **same nurse**	**Different nephrologist** (whoever is on clinics that day), d**ifferent Nurse** (whoe is on clinics that day)

11. A. Is there a policy that all patients are assessed for home dialysis? **Yes/No (Specify)**
   B. Are **ALL** patients provided dialysis modality education? **Yes/No**
   C. If **YES** to B- is it individual or group: **Individual / Group/ Combination**
   D. Is there a vascular access nurse for the renal program? **Yes/No**

12. What kinds of tools/information do you use to aid patients regarding dialysis and modality choices? ***List all resources***:
   Would you be able to provide us with a copy of such tools/information booklets/videos? **Yes/No**
13. Does your clinic have a dialysis modality coordinator? **Yes/No (get details about this person)**
14. If your clinic has a dialysis modality coordinator, do they see ALL patients who are approaching the need for dialysis? **Yes/No**
15. A. For outpatients, is there an eGFR level that generally leads to dialysis initiation? **If so, what is it?**
   B. What are the other factors that are considered important? **(list factors)**
16. Is the decision to start a patient on dialysis reviewed during multidisciplinary case rounds, or are decisions made by the physician / nurse assigned to the patient? **Team rounds / Nephrologist & Nurse / Other**
17. Does your clinic have a care pathway, clinical pathway or algorithm for managing patients with severe CKD? **Yes/No**
18. A. Does your CKD clinic/hospital have targets for home dialysis therapies (i.e. home hemodialysis and PD)? **Yes/No**
   B. Is the target for patients already on dialysis (prevalent patients) or for new dialysis patients (incident patients)? **Prevalent / incidents/ both**

C. What is the target?

19. Can you think of any improvements or changes to your program that could enhance the care of patients with CKD?

20. Any other comments you would like to make regarding CKD patient care at your clinic?
